# Innovative combination of unilateral biportal endoscopic discectomy and interlaminar dynamic stabilization using the IntraSPINE device for huge lumbar disc herniation: technical note and preliminary report

**DOI:** 10.20452/wiitm.2025.17976

**Published:** 2025-09-08

**Authors:** Zi ‑Han Fan, Jia‑Shen Shao, Hai Meng, Qi Fei

**Affiliations:** Department of Orthopedics Beijing Friendship Hospital Capital Medical University Beijing, China China

**Keywords:** huge lumbar disc herniation, interlaminar stabilization, IntraSPINE, unilateral

## Abstract

**INTRODUCTION:**

The unilateral biportal endoscopy (UBE) technique has demonstrated favorable outcomes in lumbar discectomy and decompressive laminectomy. IntraSPINE is an innovative interlaminar dynamic stabilization device providing a minimally-invasive alternative for the treatment of degenerative lumbar diseases.

**AIM:**

The objective of this study was to describe the rationale, surgical technique, and preliminary results of an innovative approach involving integration of UBE discectomy and interlaminar stabilization using IntraSPINE for the treatment of huge lumbar disc herniation (LDH).

**MATERIALS AND METHODS:**

We analyzed 5 consecutive patients with huge LDH who underwent UBE decompressive laminectomy and discectomy combined with IntraSPINE interlaminar dynamic stabilization at our hospital between May and August 2023. The IntraSPINE interlaminar spacer was implanted contralaterally to the symptomatic interlaminar space with the assistance of UBE. X-ray, computed tomography, and magnetic resonance imaging were used to evaluate the range of segmental movement, posterior disc height (PDH), and disc degeneration at the baseline, after surgery, and at the final follow-up. Clinical outcomes were assessed using the Visual Analog Scale (VAS) and the Oswestry Disability Index (ODI).

**RESULT:**

The procedure was successfully completed in all patients. Postoperative radiological imaging showed an increase in PDH and no exacerbation of lumbar disc degeneration. The VAS and ODI scores recorded after surgery and at the final follow-up visit improved remarkably, as compared with the baseline values. No surgery-related complications were reported.

**CONCLUSION:**

The combination of UBE and IntraSPINE technology demonstrated good short-term outcomes. The advantages of this hybrid approach include maintaining intervertebral height, preserving intervertebral disc structure, and minimal invasiveness.

## INTRODUCTION

Lumbar disc herniation (LDH), defined as localized displacement of intervertebral disc material beyond the typical intervertebral disc space boundary, is a common condition that causes low back pain and radiculopathy.^1^ It affects 1% to 5% of the population annually, and stands out as a primary contributor to back pain and sciatica.^2^ According to a general consensus, huge or massive LDH is defined as a condition in which the herniated disc material occupies 50% or more of the anteroposterior diameter of the spinal canal, as observed on magnetic resonance imaging (MRI).^3^;^4^ While nonsurgical care remains the cornerstone of initial treatment, discectomy is administered to effectively alleviate symptoms persisting over prolonged periods.^5^;^6^ Traditional open surgery is associated with various potential complications, including excessive loss of intraoperative vertebral side, prolonged muscle exertion, cerebrospinal fluid leakage, vertebral instability, and low back pain.^7^;^8^ To address these issues, minimally-invasive methods have gained widespread utilization in the treatment of huge LDH.^9^

One of such methods is unilateral biportal endoscopic (UBE) discectomy. It involves the use of a unilateral dual-channel endoscopic technique, typically configuring 2 channels: 1 for observation and the other for instrument operation. The observation channel commonly utilizes 0 ° or 30 ° arthroscopy.^10^ The advantages of UBE surgery include an expanded field of vision, operational flexibility, minimal invasiveness, and facilitation of comprehensive nerve decompression, promoting faster recovery.^11^ However, concerns have arisen regarding the potential risk of lumbar disc deterioration, loss of intervertebral space height, segmental instability, and LDH recurrence following decompressive laminectomy and extensive discectomy.

The IntraSPINE device (Cousin Biotech, Wervicq-Sud, France) is an innovative interlaminar dynamic stabilization device conceptualized by Giancarlo Guizzardi and introduced into clinical practice in 2007. The core material of IntraSPINE is flexible medical silica gel, while the surface material is polyester fiber. Such a composition aims to enlarge the foramina, alleviate pressure on facets and discs, and stabilize the spine without compromising its natural motion.^12^;^13^ Consequently, IntraSPINE facilitates dynamic stabilization by allowing a controlled degree of motion while protecting the lumbar intervertebral disc through the distraction of the posterior spinal elements. The device was originally indicated for the management of low back pain attributed to disc degeneration and as a postoperative intervention following lumbar discectomy.^12^

## AIM

The objective of this study was to describe the rationale, surgical technique, and the results of the innovative integration of UBE discectomy and IntraSPINE interlaminar stabilization for the treatment of huge LDH.

## MATERIALS AND METHODS

This retrospective study was carried out at our hospital from May to August 2023. A total of 5 consecutive patients were enrolled (4 men and 1 woman; mean [SD] age, 40.6 [13.6] y; range, 21–58 y), all of whom presented with huge LDH at a single level accompanied by neurological symptoms. Patient characteristics are summarized in Table 1

**Table 1:** Demographic data of the patients (n = 5)

Parameter		Value
Age, y		40.6 (13.6)
Sex	Men	4 (80)
	Women	1 (20)
Follow-up time, mo		9.5 (1.5)
Operative level L4–L5		5 (100)
Operative time, min		118.6 (13)
Blood loss, ml		57 (18.6)
Length of hospital stay, d		7.6 (0.6)
Surgery-related complications		

Data are presented as mean (SD) or number (percentage).

**Figure 1:**
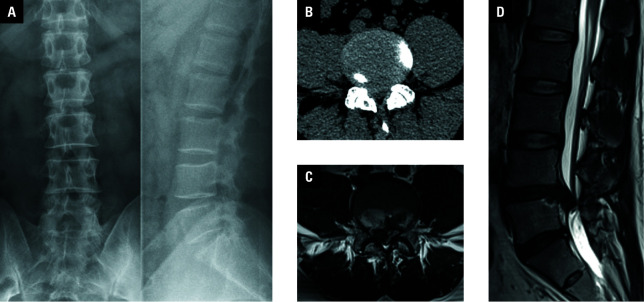
Preoperative images of a 38 -year-old man presenting with huge lumbar disc herniation (LDH) in the L4–L5 segment; A – X -ray image showing lumbar spine degeneration with narrowing of the intervertebral space at the L4–L5 level; B – computed tomography scan showing LDH in the L4–L5 segment; C, D – magnetic resonance imaging scans visualizing huge LDH occupying more than 50% of the spinal canal and causing severe compression of the dural sac and nerve roots in the L4–L5 segment

### Inclusion and exclusion criteria

The inclusion criteria were as follows: 1) age between 18 and 60 years at the time of diagnosis; 2) presence of severe low back pain and unilateral lower limb pain or numbness; 3) baseline imaging studies (lumbar X-ray, computed tomography [CT], and MRI) showing lumbar spine degeneration, narrowing of the intervertebral space, LDH, and compression of the dural sac and nerve roots (Figure 1A–Figure 1D); 4) a diagnosis of huge LDH, defined as the presence of herniated disc material occupying more than 50% of the spinal canal and causing severe compression of the dural sac and nerve roots on MRI; 5) a lack of surgical contraindications.

Patients were excluded if they met any of the following criteria: 1) age under 18 years or over 60 years, or pregnancy; 2) multilevel LDH (herniation at ≥2 lumbar levels); 3) LDH combined with other spinal pathologies, such as spinal stenosis, spondylolisthesis, spinal tumors, infections, or fractures; 4) concurrent systemic diseases that could confound neurological assessment; 5) osteoporosis; 6) absence or hypoplasia of the lumbar spinous processes.

### Ethics statement

The study was approved by the ethics committee of the Beijing Friendship Hospital, Capital Medical University (BFH20250120001). Informed consent was obtained from all participants included in the study.

### Surgical technique

#### Step 1: preoperative plan

Based on the patients’ symptoms, physical examination, and imaging findings, a diagnosis of huge LDH was established, warranting decompressive laminectomy and discectomy. To minimize operative trauma, the UBE technique was utilized for laminectomy and discectomy. In light of the potential need for performing extensive discectomy while addressing the massive intervertebral disc fragments, posing risks of postoperative intervertebral height loss, spinal instability, and accelerated disc degeneration, the decision to place the IntraSPINE interlaminar stabilization device was made preoperatively.

#### Step 2: design of surgical incisions

**Figure 2:**
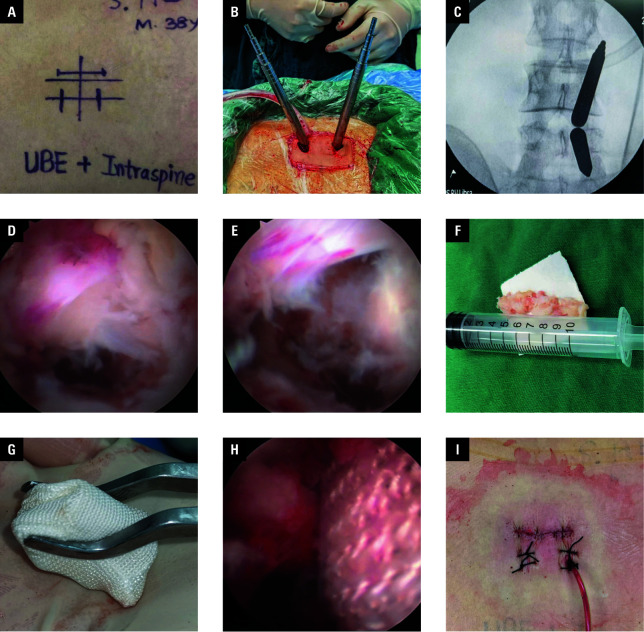
**A** – design of the incision; **B** – intraoperative picture showing 2 serial dilators utilized to progressively expand and bluntly separate the soft tissue; **C** – final position of the 2 dilators directed toward the lower lateral edge of the interlaminar space on X-ray fluoroscopy; **D** – the extruded intervertebral disc nucleus (arrow) compressing the L5 nerve root (asterisk); **E** – successful decompression of the nerve root (asterisk); **F** – the large extruded intervertebral disc nucleus in vitro (arrow); **G** – the IntraSPINE dynamic stabilizing device in vitro (arrow); **H** – the IntraSPINE dynamic stabilizing device (arrow) on endoscopy; **I** – suturing of the incisions and placement of a drainage tube

Each patient was put under general anesthesia and placed in a prone position with the abdomen draped. The target intervertebral space was identified with X-ray fluoroscopy. Before the surgery, an incision design was prepared (Figure 2A). The primary target point was positioned at the juncture of the inferior articular process of the L4 lumbar vertebra and the superior lamina of the L5 vertebra, and a horizontal line was drawn across the point. A second line was drawn along the inner edge of the L4–L5 pedicles. The incision points for observation and operation were established on the body surface along the second line, approximately 1 cm from the intersection of the 2 abovementioned lines. The incision plan included 2 transverse incisions of approximately 1 cm on the symptomatic side beside the midline for UBE access, and 1 longitudinal incision of about 3 cm on the contralateral side near the midline for placement of the IntraSPINE device. The intention was to employ the UBE technique from the symptomatic side for decompression and place the IntraSPINE device from the contralateral side.

#### Step 3: operative procedures

Two positioning needles were used to indicate the lower lateral edge of the interlaminar space from the symptomatic side of the spinous process for guiding the UBE incision. Two incisions, approximately 1 cm in length, were made in the skin and subcutaneous fascia. Following the incision, serial dilators were utilized to progressively expand and bluntly separate the soft tissue covering the bony surface (Figure 2B and Figure 2C). The 2 incisions served distinct purposes: one was used for inserting the observation endoscope, and the other for accommodating surgical instruments. Under endoscopic visualization, radiofrequency was applied to expose the surrounding anatomical structures of the interlaminar space. Subsequently, partial removal of the L4 lower and L5 upper articular processes and the upper edge of the L5 lamina was performed using a grinder, exposing the edge of the ligamentum flavum. The ligamentum flavum was excised to expose the dura and the outer edge of the nerve root (Figure 2D). A nerve retractor was then used to shield the nerve root, pulling it inward to fully remove the extruded nucleus of the intervertebral disc (Figure 2E and Figure 2F). Simultaneously, a longitudinal incision of approximately 3 cm was made along the midline, preserving the supraspinous ligament, to expose the contralateral spinous process and interlaminar space. Under endoscopic guidance, the IntraSPINE dynamic stabilization device (Figure 2G) was positioned in the interlaminar space (Figure 2H). The stability and appropriate postioning of the IntraSPINE were confirmed. Ultimately, the surgical procedure was finished with suturing of the incisions and placement of a drainage tube (Figure 2I).

### Observational index

Lumbar spine X-rays, CT, and MRI scans were obtained before and after surgery, as well as at the final follow-up visit. Radiological characteristics, including the extent of disc degeneration, level of surgical intervention, posterior disc height (PDH), and segmental range of motion, were retrospectively evaluated and recorded. Data regarding operative time, blood loss, and length of hospital stay were also collected.

Clinical outcomes were evaluated using the Visual Analog Scale (VAS) to assess back pain (VAS-B) and leg pain (VAS-L), as well as the Oswestry Disability Index (ODI). These assessments were performed on the day prior to surgery, the first postoperative day, and at the final follow-up visit. Mean follow-up duration was 9.5 months (range, 8–12 mo).

### Statistical analysis

Continuous variables were tested for normality using the Shapiro–Wilk test and are presented as mean (SD). As the data consisted of repeated measurements from the same cohort of patients across 3 time points, 1-way repeated-measures analysis of variance (ANOVA) was employed to determine the overall differences in ODI and VAS-L scores among the preoperative, postoperative, and final follow-up assessments. When the repeated-measures ANOVA indicated a significant overall effect, post-hoc pairwise comparisons were conducted using the Bonferroni-adjusted paired-samples *t* tests. The effect size is expressed as an *F* value. All statistical analyses were performed using SPSS 21.0 software (SPSS Inc., Chicago, Illinois, United States), and a *P* value below 0.05 assumed as significant.

## RESULTS

The procedure was successfully completed in all 5 patients, and no intraoperative conversion to open surgery was required. The mean (SD) operative time was 118.6 (13) minutes (range, 100–130 min), and the mean (SD) blood loss was 57 (18.6) ml. Each patient was encouraged to stand and walk with the assistance of a brace at an early stage after the operation. Radiological assessments confirmed favorable positioning of the IntraSPINE device in all cases.

Radiographic and functional outcomes are presented in Table 2. The mean (SD) VAS-L and VAS-B scores significantly decreased after surgery, reflecting alleviation of both leg and back pain. Functional outcomes were evaluated using the ODI score. In comparison with the preoperative values, the ODI scores significantly improved both early after surgery and at the final follow-up.

**Table 2:** Patient radiographic and functional characteristics before surgery, after surgery, and at the final follow-up

Parameter	Before surgery	After surgery	Final follow-up	*F* value	*P* value
ODI score, %	37.6 (2.2)	20 (4)	15.4 (4.8)	76.57	<⁠0.001
VAS-L score, points	8.4 (0.6)	1.6 (0.5)	0.4 (0.6)	620.44	<⁠0.001
VAS-B score, points	5 (1)	–	0.6 (0.6)^a^	–	<⁠0.001
PDH, mm	5.7 (1.6)	–	7.9 (1.9)^a^	–	<⁠0.001
Range of motion, °	5 (3.4)	–	4.7 (2.8)^a^	–	0.33

Data are presented as mean (SD).

**a **The follow-up values were compared with the preoperative ones.

Abbreviations: ODI, Oswestry Disability Index; PDH, posterior disc height; VAS-B, Visual Analog Scale for back pain; VAS-L, Visual Analog Scale for leg pain

**Figure 3:**
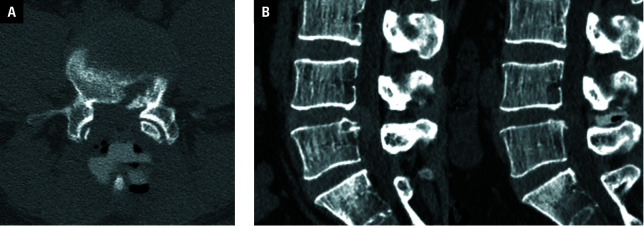
Dynamic X-ray images of a 38-year-old man with huge lumbar disc herniation before (**A**) and after surgery (**B**) showing improvement in the range of motion at the L4–L5 level from 3.5 to 3.7 °. The position of the IntraSPINE device was appropriate and stable in dynamic positions (arrow).

Postoperative CT examination confirmed an appropriate and stable placement of the IntraSPINE device at the final follow-up, demonstrating successful decompression achieved through the UBE technique (Figure 3A and Figure 3B). The anterior section of the IntraSPINE was affixed to the ligamentum flavum, effectively expanding the interlaminar space. The mean (SD) PDH at the L4–L5 level was 5.7 (1.6) mm at baseline, and improved significantly to 7.9 (1.9) mm at the final follow-up Table 2.

**Figure 4:**
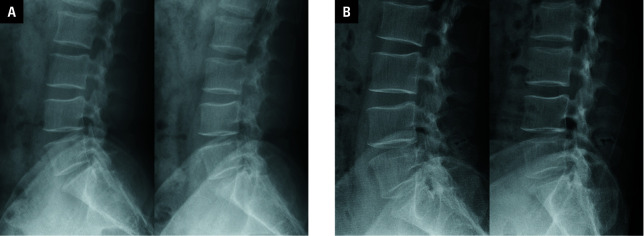
Dynamic X-ray images of a 38-year-old man with huge lumbar disc herniation before (**A**) and after surgery (**B**) showing improvement in the range of motion at the L4–L5 level from 3.5 to 3.7 °. The position of the IntraSPINE device was appropriate and stable in dynamic positions (arrow).

A dynamic X-ray examination also showed stable positioning of the IntraSPINE at the final follow-up Figure 4. The mean (SD) range of motion at the L4–L5 level was 5 (3.4) °, and it was similar to the preoperative range (mean [SD], 4.7 [2.8] °; *P *= 0.33; Table 2), suggesting preservation of movement in the operative segment. No cases of postoperative instability in the L4–L5 segment were observed (Figure 4A and Figure 4B).

**Figure 5:**
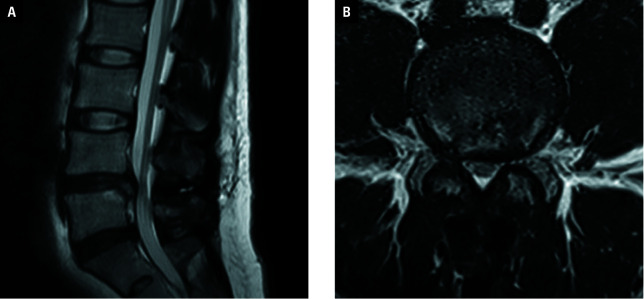
Postoperative magnetic resonance images of a 38-year-old man with huge lumbar disc herniation at the final follow-up; **A** – sagittal view showing satisfactory decompression and stable placement of the IntraSPINE device (arrow), with no exacerbation of intervertebral disc degeneration at the L4–L5 level; **B** – axial view showing satisfactory decompression and stable placement of IntraSPINE (arrow)

The final follow-up MRI indicated satisfactory decompression, with no exacerbation of intervertebral disc degeneration observed at the L4–L5 level (Figure 5A and Figure 5B), highlighting the positive role of the IntraSPINE device in lumbar disc protection. The position of the IntraSPINE at the L4–L5 segment remained stable. None of the patients experienced symptom recurrence.

## DISCUSSION

Huge LDH is characterized by herniated disc material occupying 50% or more of the anteroposterior diameter of the spinal canal on MRI. Should clinical symptoms endure despite conservative treatment, surgical intervention becomes imperative. Recently, there has been a notable surge in the preference for minimally-invasive spine surgery in the treatment of LDH, marking a significant shift from conventional open discectomy toward endoscopic procedures.^14^ The UBE technique represents an innovative minimally-invasive endoscopic method designed for addressing spinal disorders. ^15^ initially documented the adoption of this technique for the treatment of LDH in 1996.

The UBE procedure primarily entails creation of 2 channels on a single side of the spinal area. One of these channels functions as the entry point for the optical instrument and irrigation system, while the second accommodates the surgical instruments used for laminectomy or discectomy. To establish the working space, only a small extent of soft tissue is stripped away from the interlaminar space using serial dilators, a bipolar radiofrequency probe, and continuous saline irrigation through the channels. The lens and instruments can be precisely maneuvered through the soft tissue channels to access the target area. The vision of the surgical field is expanded with different channels, resulting in reduced vascular bleeding under continuous irrigation. Consequently, this method facilitates clear visualization of neural elements, surrounding soft tissues, and vascular and bony structures.^16^ The procedural steps for decompression and discectomy, as well as the primary instruments used in UBE, closely resemble those employed in conventional open posterior lumbar operations. Therefore, practitioners with experience in open spinal surgery can adeptly perform UBE after only a brief training period.^17^ The learning curve of UBE is also relatively flat and short.^18^ Moreover, the working portal does not restrict or limit the operating instruments of UBE. Larger surgical instruments typically utilized during conventional operation, such as an osteotome, rongeur, forceps, and nerve retractor, can be used to improve the working efficiency.^14^

In the patients included in our study, the nucleus of the lumbar intervertebral disc was significantly extruded into the spinal canal, leading to severe compression of the dural sac and nerve root. Due to the failure of conservative treatment, we decided to perform decompression or discectomy via the UBE technique to minimize the surgical wound. Because of huge extrusion of the lumbar disc, the surgeries were expected to be challenging. It was anticipated that in order to maximize the extraction of the extruded nucleus causing nerve compression, excessive removal of the lamina might be required, potentially leading to damage to the facet joints. Additionally, there existed a risk of dural tear during the excision of the extruded intervertebral disc. Fortunately, all UBE procedures were completed successfully without dural tear, and decompression was achieved in all cases.

Although endoscopic removal of the nucleus pulposus has been shown to achieve satisfactory therapeutic effects, there is still a possibility of recurrence after surgery.^19^ The main reason for recurrence is the significant reduction in intervertebral space after aggressive discectomy, leading to instability in the operative segment, which causes vertebral slippage and accelerates degeneration.^20^ The management of huge LDH has been associated with worse outcomes in long-term follow-up. Internal disc derangement, resulting from a substantial loss of nucleus pulposus accompanied by a significant annular tear, is an important contributor to chronic back pain.^21^

In patients with huge LDH, massive removal of the intervertebral disc nucleus results in an increased risk of intervertebral disc collapse, potentially hastening postoperative instability and degeneration of the intervertebral disc in the surgical segment. To prevent this, we decided to implement the IntraSPINE interlaminar dynamic stabilization device after the UBE procedure.

Interlaminar stabilization devices have been used to treat disc or nucleus pulposus herniation and segmental instability since 1958.^22^;^23^ They have proven efficacy in restoring intervertebral height, enlarging the foramina, alleviating pressure on facets and discs, and stabilizing the spine without compromising its natural motion.^24^;^25^;^26^ Additionally, their implantation is relatively straightforward, with short operative time and minimal blood loss.^24^;^27^ Therefore, placement of an interlaminar stabilization device was found to be safe and effective after decompression.^26^;^28^ The device is usually placed between 2 adjacent spinous processes after surgical decompression, with flanges on the superior and inferior aspects of the device anchoring to the superior and inferior spinous processes, respectively.^26^ The IntraSPINE device distinguishes itself from conventional interspinous implants, such as the X-stop (Francis Medical, Maple Grove, Minnesota, United States) and Bacfuse (Evergen, Alachua, Florida United States), by its elastic rather than rigid nature. Notably, it exhibits diverse compression ratios between its anterior and posterior components, with the anterior segment filled with medical silica gel, and the posterior segment remaining hollow. The expansion of the interlaminar space is predominantly facilitated by the anterior component of IntraSPINE, strategically positioned between the remaining portion of the laminae in proximity to the ligamentum flavum. Such positioning, closer to the instant axis of rotation and the sagittal axis of the facet joints, effectively reduces stress on the facet joints and intervertebral discs.^13^;^29^ Meanwhile, the device’s posterior component chiefly contributes to dynamic stability. Essentially, IntraSPINE was shown to improve segmental instability, help uphold sagittal balance, and restore the natural movement of the treated spinal segment.^30^ Moreover, promising outcomes have emerged from studies employing IntraSPINE in the treatment of degenerative disc disease, confirming its effectiveness in halting or even reversing gradual disc degeneration.^12^ The indications for using IntraSPINE, according to Guizzardi,^31^ included low back pain caused by disc degeneration, lumbar instability, post-lumbar discectomy status, and chronic low back pain caused by zygapophyseal joint syndromes, among others.

In our study, while placing the IntraSPINE device, we carefully removed a minimal portion of the perispinal muscles and were cautious to preserve both the lamina and posterior ligamentous structures from the contralateral incision. Additionally, we only slightly disrupted the interspinous ligament, aiming to minimize the surgical trauma associated with the procedure.

Effective utilization of this innovative combination of 2 minimally-invasive techniques (interlaminar stabilization coupled with decompressive laminectomy and discectomy) yielded satisfactory short-term outcomes. The patients’ symptoms were alleviated immediately after surgery, while the VAS and ODI scores improved significantly during the follow-up. As anticipated, the intervertebral height of the operative segment was maintained, further lumbar intervertebral disc degeneration was effectively prevented, and mobility of the operated segment was preserved without segmental instability.

### Limitations

Some limitations of this study need to be acknowledged. We attempted to evaluate the feasibility of combining 2 minimally-invasive techniques in treating LDH. Although the outcomes are encouraging, a multicenter, prospective, randomized study with a larger sample size is warranted to confirm our findings. The hybrid operations were performed for the first time in this research, so the degree of proficiency needs to be improved for further development and application of this new technique. A longer follow-up is also needed to verify the long-term efficacy of the operation.

## CONCLUSIONS

The main advantages of performing a combination of UBE discectomy and IntraSPINE interlaminar device placement for huge LDH include: 1) safe and reliable therapeutic efficacy, 2) effective preservation of the intervertebral height and lumbar disc, 3) preserved mobility of the operative segment without segmental instability, and 4) adherence to the concept of minimally-invasive surgery.
